# Examining the Existence of Cognitive Thresholds in Highly Quantitative College Courses

**DOI:** 10.3390/jintelligence12040037

**Published:** 2024-03-26

**Authors:** You Zhou, Nathan R. Kuncel, Paul R. Sackett

**Affiliations:** Department of Psychology, University of Minnesota-Twin Cities, Minneapolis, MN 55455, USA; kunce001@umn.edu (N.R.K.); psackett@umn.edu (P.R.S.)

**Keywords:** quantitative ability, cognitive thresholds, college performance

## Abstract

While the dominant finding indicates a monotonic relationship between cognitive ability and academic performance, some researchers have suggested the existence of cognitive thresholds for challenging coursework, such that a certain level of cognitive ability is required for reaching a satisfactory level of academic achievement. Given the significance of finding a threshold for understanding the relationship between cognitive ability and academic performance, and the limited studies on the topic, it is worth further investigating the possibility of cognitive thresholds. Using a multi-institutional dataset and the necessary condition analysis (NCA), we attempted to replicate previous findings of cognitive thresholds on the major GPA of mathematics and physics-majored students, as well as the course grade of organic chemistry, to examine whether high SAT math scores constitute a necessary condition for obtaining satisfactory grades in these courses. The results from the two studies do not indicate an absolute cognitive threshold point below which students are doomed to fail regardless of the amount of effort they devote into learning. However, we did find that the chance of students with a low level of quantitative ability to succeed in highly quantitative courses is very small, which qualifies for the virtually necessary condition.

## 1. Introduction

The relationship between cognitive ability and academic performance has received considerable attention from both educational researchers and college admission decision makers. Past research on cognitive ability and academic outcomes suggests a monotonic relationship between the two (Arneson et al. 2011; O’Connell 2018), and the monotonicity holds even for gifted students who are in the top 1% of cognitive ability (Robertson et al. 2010). Although monotonicity does not equate linearity, as the former indicates two variables changing in the same direction whereas the latter also requires the relationship to change at a constant rate (Arneson et al. 2011), previous findings support the notion that cognitive ability positively and continuously predicts academic performance throughout the ability continuum (Brown et al. 2021). However, some researchers have suggested the possible existence of cognitive thresholds for very difficult coursework, where if students score below a certain point on cognitive ability, they will perform poorly regardless of whether or not they generally achieve good grades in other disciplines (Hsu and Schombert 2010). The finding of a monotonic relationship between ability and overall measures such as GPA, which is a composite of grades across a variety of courses, does not preclude the possibility of a cognitive threshold for certain individual courses. Hsu and Schombert (2010) and Tynan et al. (2020) have provided preliminary evidence for the existence of cognitive thresholds, yet these are the only two studies that have looked at this effect so far.

### 1.1. Cognitive Thresholds

On average, higher admission test scores are associated with higher performance at school (Julian 2005; Sawyer 2013). However, this monotonicity does not indicate that individuals below a certain point of cognitive ability cannot or will not succeed academically. For example, Westrick et al. (2019) divided students into five SAT score ranges and report that among students with a HSGPA of 3.0, those in the top SAT score category have over a 90% probability of reaching the 2.5 GPA threshold while those in the bottom SAT category have a 40% probability of doing so. Thus, although test scores are predictive of performance on average, some lower-scoring individuals do indeed perform well.

While the monotonic relationship holds for overall performance, some studies suggest that there are domains in which the relationship is more absolute. For example, Hsu and Schombert (2010) have examined the idea that high GPA or mastery of course content become very unlikely to happen below a certain SAT threshold using a large academic record dataset of undergraduates at a northwestern university. Their results indicated that cognitive thresholds were exhibited in a few majors that usually demand a high level of quantitative reasoning such as mathematics and physics, such that no student with an SAT math score of less than roughly 600 earned a GPA higher than 3.5 in physics and math classes. This study provided initial evidence for a minimum cognitive threshold required for a high level of performance in physics and math classes.

In examining the necessary conditions for achieving academic success, Tynan et al. (2020) have found that attendance, high school GPA, and cognitive ability, as indexed by the ACT, showed the strongest necessary-but-not-sufficient effect for achieving high grades in specific classes as well as overall GPA. In other words, high scores on these three variables not only increase the likelihood of achieving academic success, but they may also be the prerequisites for obtaining a very high course grade and GPA. Therefore, this study provided empirical support for a cognitive threshold on academic performance, as only when the cognitive ability level is above the threshold value is it possible to achieve a desired level of academic success.

### 1.2. Theoretical Arguments for and against Cognitive Thresholds in Academic Performance

In addition to the empirical evidence, there are also theoretical arguments for the necessity of cognitive ability in achieving academic success. In the academic performance literature, numerous studies have provided support for cognitive ability and high school grades being the strongest predictors of academic achievement (e.g., Arneson et al. 2011; Roth et al. 2015; Westrick et al. 2015). In their meta-analysis of the relationship between cognitive ability and school performance, Roth and colleagues found a corrected mean correlation of 0.54 between the two variables, which led them to conclude that cognitive ability is a substantial prerequisite for scholastic success measured through course grades. In their moderator analyses, Roth et al. (2015) found that the mean corrected correlation was highest in the mathematics and science (ρ = 0.49) subjects compared to other subjects such as languages (ρ = 0.44) and social sciences (ρ = 0.43). The authors attributed the differences in validities to subject content. Language and social science classes typically require memorizing content and facts, which makes motivation a more important factor in determining course grades in these classes than in math and science classes, where students must predominantly understand content.

Despite the promising initial results for cognitive thresholds, there is also the possibility of not finding a threshold in some cases. It is of little dispute that academic performance is influenced by many factors, both intellective and non-intellective ones. For example, Credé and Kuncel (2008) argued that study habits and study skills represent the third pillar supporting academic performance, as they found incremental validity of study habits and skills in predicting current grades beyond standardized tests and previous grades. Additionally, motivational variables such as personality, academic self-efficacy, and need for achievement have also been found to be related to academic performance at all levels and are independent of cognitive factors (Poropat 2009; Hessen and Kuncel 2022; Honicke and Broadbent 2016; Krau 1982; Meier et al. 2014). This means that although a student with less quantitative skills may be at a disadvantage in a high-quantitative course, sufficient study skills and motivation can compensate, at least to some degree.

Second, in college, students usually self-select into classes, especially for very advanced coursework. In the course of taking elementary classes, students obtain feedback regarding their performance on the given subject, which helps them form an estimate of their ability levels in comparison to the levels required to succeed in classes. Those with a low perception of their ability levels are likely to change majors or avoid certain topics (Malgwi et al. 2005; Musu-Gillette et al. 2015). As a demonstration, Zhang et al. (2020) found that only 16.5% of students entering college with a pre-med goal subsequently completed all coursework required for medical school entry, and attrition rates were highest when taking elementary classes and declined as students took more advanced classes. Therefore, we might not see cognitive ability being a prerequisite for obtaining good grades in highly difficult classes, as only those with high cognitive ability survived the attrition process, which could attenuate the relationship between cognitive ability and academic performance found in those classes. Similarly, the lower-ability members of the self-selected group who do proceed onto more difficult coursework may be especially motivated and have study habits and skills that allow them to obtain good grades (Robbins et al. 2009). This scenario does not mean that a threshold does not, in theory, exist but that we will not observe them in applied settings.

### 1.3. The Current Study

Although two studies (Tynan et al. 2020; Hsu and Schombert 2010) have examined the necessity of cognitive ability in achieving academic success and produced promising results, it is worth exploring the phenomenon further for multiple reasons. First, providing evidence for a cognitive threshold in obtaining good grades is a key finding as it carries great theoretical and practical significance. As replication of prior studies can lead to the accumulation of reliable findings on important topics, the current study seeks to replicate findings from the previous two studies with a larger and more nationally representative sample. Second, the datasets of both Tynan et al. and Hsu and Schombert came from single-institution samples, which limits the generalizability of their findings to a broader population as the curriculum and course difficulty can vary from one college to another. By using a large dataset with a multi-institutional sample, we are able to examine if there are indeed cognitive thresholds below which no matter how hard the student works, the prospect of obtaining good grades remains slim. The results of this study would have high generalizability as the dataset contains more than 100 universities in the country.

Lastly, while the findings from Hsu and Schombert (2010) suggest that there may be thresholds for very difficult coursework in physics and mathematics majors, their conclusion was not based on formal analyses beyond a scatterplot. Although a scatterplot is informative in helping readers visualize the threshold phenomenon, it would be useful to conduct a statistical analysis that could quantify the degree of necessity of quantitative reasoning in helping physics and mathematics students obtain good grades. This goal can be achieved by employing Dul’s (2016) necessary condition analysis (NCA), which allows researchers to determine whether an independent variable represents a necessary-but-not-sufficient condition for a given outcome.

### 1.4. An Overview of NCA

Although both can imply causation, necessity and sufficiency are two totally different concepts (Bindra et al. 1980). Statements indicating that a variable X is sufficient for Y (e.g., “If X, then Y”) imply that a sufficient cause X ensures the existence of the outcome Y. On the other hand, statements such as “No Y without X” indicate that cause X is necessary for the outcome Y to exist. Namely, an outcome can only exist after its necessary cause has taken place, and the outcome will not exist without the cause (Dul 2016). It is crucial to understand the necessary conditions of important outcomes such as academic success to prevent guaranteed failure and increase the probability of successfully achieving the outcome, yet they cannot be tested using traditional data analysis approaches such as correlation or multiple regression as these approaches focus on factors that could increase the occurrence of a given outcome, whereas a necessary-but-not-sufficient condition serves as a constraint that prevents an outcome from occurring. Such a constraining effect can be examined using NCA, developed by Dul.

For example, the relationship between two continuous variables X and Y can be visualized through a scatterplot. If a necessary-but-not-sufficient relation exists between the two variables, there should be a notable absence of data points in the upper left corner of the plot. The empty space indicates the constraining effect of X on achieving a desired level of Y. Namely, no instance can be found that has both a high level of Y and a low level of X, which thus suggests that reaching a specific X value is required to obtain a desired level of Y. The size of the empty space is used by NCA to calculate an effect size representing the degree of necessity of X on Y. In the analysis, a ceiling line is drawn on the scatterplot such that there is no data point above the line. The ceiling line can be drawn in several ways depending on whether variables are discrete or continuous (Dul 2016). The space above the ceiling line is referred to as the ceiling zone, and the area where data points are present is referred to as the empirical scope. The effect size of the necessary condition (*d*) is the area of the ceiling zone divided by the area of the empirical scope, and it ranges from 0 to 1. In his introductory paper on the analysis, Dul proposed some preliminary effect size interpretations, such that 0 < *d* < 0.10 is considered a small effect, 0.10 < *d* < 0.30 a medium effect, 0.30 < *d* < 0.50 a large effect, and d ≥ 0.50 a very large effect. However, Dul also suggested that the interpretation of the effect size depends on the specific context.

**Figure 1:**
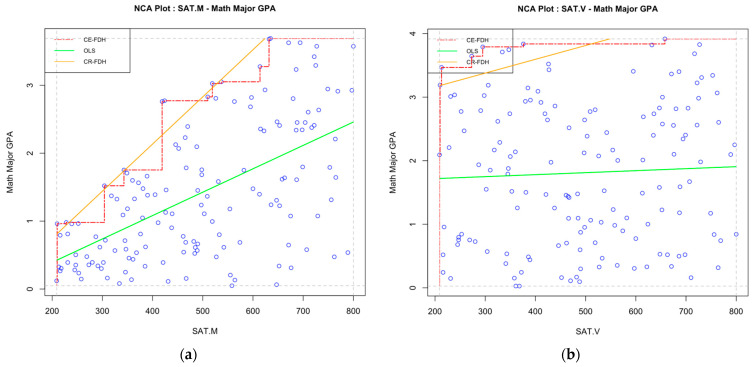
(**a**) Simulated necessary condition analysis on the relationship between SAT math scores and major GPA in students majoring in mathematics. OLS = Ordinary least squares regression line. CE-FDH = Ceiling envelopment-free disposal hull. CR-FHD = Ceiling regression-free disposal hull^1^. NCA effect size (CR-FDH) *d* = 0.30. (**b**) Simulated necessary condition analysis on the relationship between SAT verbal scores and major GPA in mathematics. *Note*: *N* = 150. “SAT.V” = SAT-verbal score. “SAT.M” = SAT-math score. “Math Major GPA” = major specific GPA for students majoring in mathematics. OLS = Ordinary least squares regression line. CE-FDH = Ceiling envelopment-free disposal hull. CR-FHD = Ceiling regression-free disposal hull. NCA effect size (CR-FDH) *d* = 0.03, *p* < 0.001.

To serve as a demonstration, we created a simulated dataset modeled on the illustration of NCA provided by Tynan et al. (2020). In this hypothetical case, we investigate whether SAT math and verbal scores constitute a necessary condition for students to obtain a high math major GPA, with the results shown in Figure 1. In both scatterplots, the NCA package shows two ceiling lines, one linear and one discrete; our interest is in the linear ceiling line as our SAT predictors can be viewed as continuous variables. The ceiling lines were drawn to separate the ceiling zone and the empirical scope, and the effect size was calculated as the ratio of the sizes of the two areas. Looking at the two plots, it is obvious that there is a much larger ceiling zone on the scatterplot between SAT math scores and math GPA than on the one between SAT verbal scores and math GPA. Additionally, the effect size is *d* = 0.30 for Figure 1a and *d* = 0.03 for Figure 1b. Therefore, SAT math scores represent a much stronger necessary-but-not-sufficient condition for high math GPA when compared to SAT verbal scores. Specifically, high math GPA is only possible when scores on the quantitative section of the SAT are also high. For example, the plot indicates that no math-major student with SAT math scores below 500 obtained a major GPA higher than 3.

Therefore, through conducting NCA between SAT math scores and students’ grades in highly quantitative classes, we are able to provide an effect size estimate that addresses the problem of whether there exists a cognitive threshold below which high course grades are very unlikely to be obtained no matter how hard the student works. Such an estimate is not only conducive in developing a more complete understanding of the relationship between intelligence and academic performance, but it also carries practical utility for educational counseling purposes, where students could receive advice regarding the implications of their course taking so as to avoid attaining poor grades and scholastic probation.

Hsu and Schombert (2010) have found preliminary evidence that suggests the existence of a cognitive threshold in mathematics and physics majors. In Study 1, we attempted to replicate their findings in our multi-institution dataset using NCA, and then in Study 2 we examined the existence of cognitive thresholds in a single class with a reputation for difficulty, namely, organic chemistry. In this paper, we hope to provide a more thorough investigation of the cognitive threshold phenomenon through examining whether we could replicate previous findings regarding the existence of such a threshold and also whether thresholds exist in obtaining good grades in a very difficult course.

## 2. Study 1 Method

### 2.1. Sample

The sample used for this study was a subset of the data collected by The College Board for freshman cohorts from 2006 to 2009, which contains 392,135 students in total. In order to ensure the comparability of GPA score across different schools, we only included schools that used a GPA and course grading system that ranges from 0 to 4 (i.e., F = 0.0, D = 1.0, C = 2.0, B = 3.0, A = 4.0). Moreover, we only included students who still majored in mathematics and physics at their fourth year of college. After removing students from schools that used a different GPA system, as well as students with missing data for either in-major GPA or SAT math score, the final dataset contained 31 schools and 628 students with a mathematics major, and 31 schools and 526 students with a physics major. In the math-major sample, 40.8% was female, and the ethnicity breakdown was 89.6% Caucasian, 1.8% African American, 3.7% Asian or Pacific Islander, 0.3% American Indian/Alaskan Native, 2.2% Hispanic, Mexican, or Puerto Rican, and 2.8% Other. In the physics-major sample, 22.2% is female, and the ethnicity breakdown was 85.5% Caucasian, 1.2% African American, 3% Asian or Pacific Islander, 0.1% American Indian/Alaskan Native, 4.2% Hispanic, Mexican, or Puerto Rican, 6.1% Other.

### 2.2. Measures

*SAT math scores.* Only the SAT math subtest was used in this study, with possible scores ranging from 200 to 800.

*College major.* Students’ major information in their fourth year were obtained from college records.

*In-major GPA.* The major-specific GPA was calculated as the average of grades obtained in all major classes. For example, the in-major GPA for a math-major student is the average of all the math classes the student has taken in four years.

### 2.3. Analyses

The goal of this study was to replicate the finding of Hsu and Schombert (2010) that no physics and mathematics major student with a SAT math score less than 600 was able to obtain an in-major GPA higher than 3.5. To achieve this goal, NCA was conducted on the relationship between math- and physics-majored students’ SAT math scores and their in-major GPA. The analyses were conducted using Dul’s (2016) NCA package on R (Version 2022.07.1, R Core Team 2022).

## 3. Study 1 Results

SAT math scores (math dataset: mean = 698.22, standard deviation = 72.34; physics dataset: mean = 698.35, standard deviation = 72.07) correlates with in-major math GPA (mean = 3.20, standard deviation = 0.61) at 0.38, and with in-major physics GPA at 0.30 (mean = 3.25, standard deviation = 0.67).

In terms of a simple descriptive analysis, a close inspection of the dataset indicates that only two math-major students (out of 628 math students) and nine physics-major students (out of 528 physics students) who scored below 600 on SAT Math and obtained an in-major GPA higher than 3.5. Hsu and Schombert’s (2010) study found that no mathematics and physics student with an SAT math score of less than roughly 600 earned an in-major GPA higher than 3.5. In the current dataset, although there were two math-major and nine physics-major students who scored below 600 on SAT math yet obtained an in-major GPA above 3.5, we believe the result is close enough to conclude that the Hsu and Schombert’s finding of a cognitive threshold was replicated as the number of students breaking the threshold was very small in both majors.

**Figure 2:**
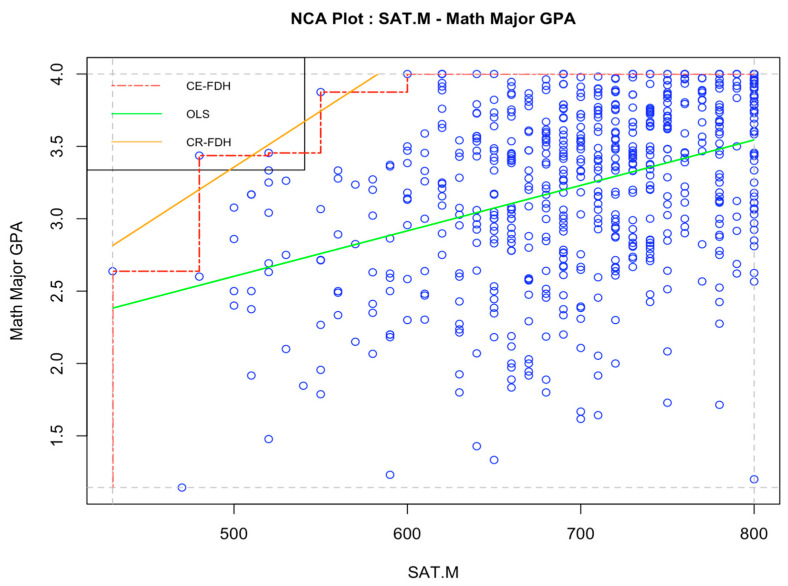
Necessary condition analysis on the relationship between SAT math scores and major GPA in mathematics, Study 1. *Note*: *N* = 628. “SAT.M” = SAT math score with final zero dropped. “Math Major GPA” = major specific GPA for students majoring in mathematics. OLS = Ordinary least squares regression line. CE-FDH = Ceiling envelopment-free disposal hull. CR-FHD = Ceiling regression-free disposal hull.

**Figure 3:**
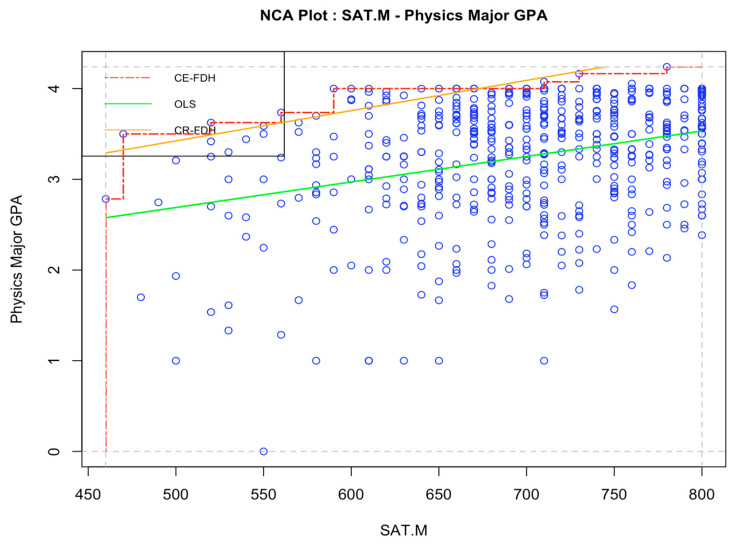
Necessary condition analysis on the relationship between SAT math scores and major GPA in physics, Study 1. *Note*: *N* = 526. “SAT.M” = SAT math score with final zero dropped. “Physics Major GPA” = major specific GPA for students majoring in physics. OLS = Ordinary least squares regression line. CE-FDH = Ceiling envelopment-free disposal hull. CR-FHD = Ceiling regression-free disposal hull.

The results of the NCA analysis of the relationship between SAT math scores and math major GPA is plotted in Figure 2, and the relationship between SAT math scores and physics major GPA is plotted in Figure 3. The effects sizes obtained suggest that SAT math scores are a weak necessary-but-not-sufficient condition for obtaining a high major GPA in mathematics (*d* = 0.09 using the CR-FDH designed for continuous variables) and physics (*d* = 0.09, also using CR-FDH). The effect sizes obtained from the two ceiling lines are very similar, both indicating a small constraining effect of SAT math scores on obtaining high in-major GPA for both mathematics and physics students.

## 4. Study 1 Discussion

In this study, we attempted to replicate Hsu and Schombert’s (2010) finding of a cognitive threshold for mathematics and physics majors with a multi-institution dataset and a new analysis approach called NCA, which allowed us to test the necessary-but-not-sufficient relation between quantitative ability and high math and physics-major GPA, and the results suggested small effect sizes (*d* = 0.09) for both majors. Despite the small effects, we believe that Hsu and Schombert’s finding was replicated in the current study due to two reasons. First, the number of students who obtained an SAT math score of less than roughly 600 and subsequently earned an in-major GPA higher than 3.5 was negligible in both majors (0.3% and 1.7% for math and physics students, respectively), while many students with higher scores earned that GPA or higher. It is possible that the cases with a 3.5 GPA or better and scores less than 600 are due to random error (e.g., abnormally poor performance on SAT math due to not feeling well on the test day). Therefore, even though the chance is not zero, it is very unlikely for math and physics students with a low SAT math score to obtain a high in-major GPA at the end of their fourth year. Second, in his introductory paper on the analysis, Dul (2016) noted the possibility of a “virtually necessary condition”, in which there is “almost always” or “usually” failure under the absence of a necessary condition; he proposes a ceiling line be drawn such that 95% of cases lie below it. We believe the small percentages listed above suggest quantitative reasoning ability to be a virtually necessary condition of in-major GPA for math- and physics-major students.

One limitation of Study 1 might be our concentration on mathematics and physics majors as we attempted to replicate the findings from Hsu and Schombert’s (2010); thus, it remains unclear whether cognitive thresholds exist in other highly quantitative majors such as chemistry and statistics. Moreover, although we used in-major GPA as the outcome, which controlled for the possibility of students taking less difficult elective courses to boost their GPAs, the calculation of in-major GPA still includes grades from many less advanced math courses. As a result, the difficulty of obtaining an in-major GPA of 3.5 is lower than of obtaining good grades in those very hard courses. Therefore, in Study 2, we investigated the cognitive threshold of academic performance by examining its existence in one of the most challenging classes in the college science curriculum.

### Study 2

The goal of Study 2 was to examine the existence of a cognitive threshold in classes with a high quantitative load, and we decided to focus on organic chemistry classes. Organic chemistry has often been cited as a “decision point” for students planning a career in a competitive science discipline, such as medicine, biochemistry, or biomedical engineering. Although the highest attrition rate of pre-med students was found in more elementary chemistry classes (Zhang et al. 2020), organic chemistry is still widely considered to be a challenging course in the college science curriculum, with significant failure rates (Grove and Bretz 2010; Turner and Lindsay 2003; Crimmins and Midkiff 2017) and a requirement of a high level of complex and multivariate reasoning, abstract and analytic thinking, and problem-solving ability (Lieber and Graulich 2020; Graulich 2015). The difficulty of the course makes it most useful for the examination of the cognitive threshold phenomenon, as obtaining good grades demands a high level of quantitative reasoning, which could result in a cognitive threshold on class performance.

## 5. Study 2 Method

For this study, we used a different subset of the College Board data used in Study 1. We focused on 48,116 students who are enrolled in organic chemistry at 141 U.S. colleges and universities. The colleges were heterogeneous with respect to selectivity, institution size, regional location, and public vs. private control. Sample sizes for each college ranged from 1 to 2682 (*M* = 305, *SD* = 514). In the current sample, 56.4% was female, and the ethnicity breakdown was 59.1% Caucasian, 5.36% African American, 21.4% Asian or Pacific Islander, 0.4% American Indian/Alaskan Native, 6.7% Hispanic, Mexican, or Puerto Rican, 3.3% Other.

To help standardize the course content across different universities, we selected only introductory organic chemistry, or the first class in the organic chemistry series (i.e., organic chemistry I) and we excluded all the labs as they have very different grading policies and course content from the lectures. Given that organic chemistry is a very challenging class, many students took the class more than once to obtain a satisfactory grade. For those students, we only kept the grade they received from their first attempt at taking the class, as that grade would be less contaminated by practice and retest effects. After removing students with missing data on either SAT math or course grade in the organic chemistry class, the final dataset for this analysis contained 130 schools and 47,238 students.

### 5.1. Measures

*SAT math scores.* Only the SAT math subtest was used in this study, with possible scores ranging from 200 to 800.

*Course grades.* Individual course grades for introductory organic chemistry were reported by the schools. The course grades ranged from 0 to 4 (i.e., F = 0.0, D = 1.0, C = 2.0, B = 3.0, A = 4.0).

### 5.2. Analyses

We first compiled descriptive statistics, showing the percentage of students at various SAT levels who reached various grade thresholds, and then we conducted NCA using Dul’s (2016) package on R (Version 2022.07.1).

## 6. Study 2 Results

The correlation between SAT math score (mean = 631.43, standard deviation = 85.48) and the course grade of organic chemistry (mean = 2.59, standard deviation = 1.07) is 0.31. Unlike Study 1, in which we had specific numbers for the threshold (i.e., SAT math scores below 600), Study 2 is exploratory, and we do not have a standard for testing both SAT math scores and course grades against the threshold hypothesis. Therefore, we investigated various combinations of math scores and course grades as well as the number of students that fall into each category (Table 1). Table 1 indicates that although there does not seem to be a clear-cut threshold point for SAT math scores below which no student in the group could obtain a course grade of 4.0, the likelihood of students scoring less than 400 or between 400 and 500 on SAT math to obtain a course grade higher than B+ is very small (SAT math score < 400: 1% obtained B+ and A−, 3% for A; SAT math score between 400 and 500, 4% obtained B+, 2% obtained A−, and 6% obtained A), and it is evident from the table that it is increasingly less likely for students with a low SAT math score to obtain a high grade in organic chemistry.

**Figure 4:**
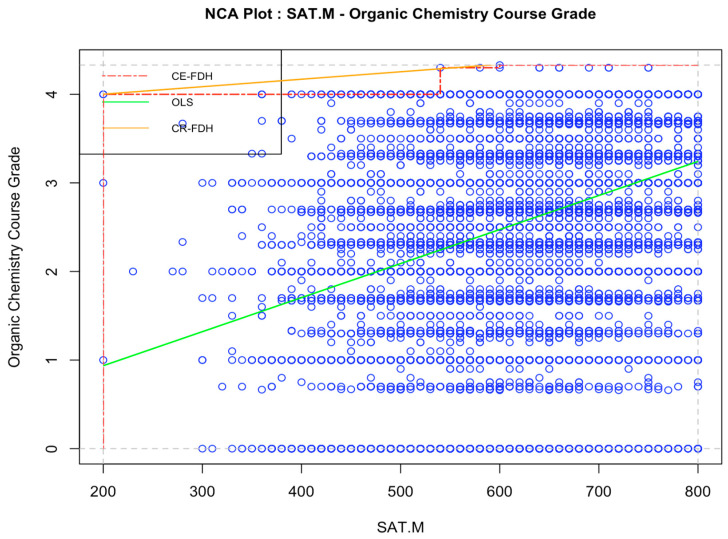
Necessary condition analysis on the relationship between SAT math scores and organic chemistry course grade, Study 2. *Note*: *N* = 48,119. “SAT.M” = SAT math score with final zero dropped. OLS = Ordinary least squares regression line. CE-FDH = Ceiling envelopment-free disposal hull. CR-FHD = Ceiling regression-free disposal hull.

The NCA results of the relationship between SAT math scores and the course grade in organic chemistry are plotted in Figure 4. The effect size measures suggest that SAT math scores are a weak necessary-but-not-sufficient condition for obtaining a high grade in organic chemistry (*d* = 0.03).

## 7. Study 2 Discussion

By employing descriptive statistics and NCA, we examined the necessary-but-not-sufficient relation between SAT math scores and high course grades in organic chemistry, a very challenging class in the college science curriculum (Grove and Bretz 2010). The NCA results indicate a small effect size (*d* = 0.03), suggesting that high SAT math scores are a weak necessary condition of earning satisfactory grades in an organic chemistry class. We also looked at the specific percentages of students in each combination of SAT math scores and course grades; these percentages do not indicate a clear cut-off point of SAT math scores below which no student would be able to achieve a satisfactory course grade (i.e., B and above), yet they show a decreasing likelihood of students with lower SAT math scores obtaining good grades in the class and a very small chance of students with SAT math scores of less than 500 obtaining satisfactory grades (i.e., B+ or above). These results indicate that while there does not exist an absolute cognitive threshold for performance in the organic chemistry class, quantitative ability might be a virtually necessary condition for the course performance, such that it is still possible for students who scored relatively low on SAT math to earn a good grade in the class; however, the likelihood is very low, as only 14 students (out of 277) who scored below 400 and 311 (out of 2906) students with a score between 400 and 500 on SAT math were able to obtain a grade higher than B at the end of the class.

Similar to Study 1, one shortcoming of this study is the sole focus on one class. Thus, the generalizability of the current study’s findings to other classes is unclear until it is examined in future studies. Moreover, in examining the relationship between course grades and SAT math scores, we only used grades from the lecture classes and excluded those from the lab sections. However, for some schools, grades obtained in lab sessions are included in the calculation of the final course grade, whereas for others, lab sessions are treated as a separate class and their grades only count toward GPA calculation, which could result in incomparability of the course grades in terms of the calculation of course grades.

## 8. General Discussion

While the results from previous studies tend to support a monotonic relationship between cognitive ability and academic performance across the whole ability continuum (Arneson et al. 2011; Robertson et al. 2010), some researchers have suggested the potential existence of cognitive thresholds for very difficult coursework, such that a certain level of cognitive ability is a prerequisite for reaching a satisfactory level of academic achievement (Hsu and Schombert 2010; Tynan et al. 2020). These two findings do not necessarily conflict with each other, as previous studies on the relationship between ability and academic performance usually employ overall measures of performance, such as GPA, that aggregate grades across a variety of courses, whereas cognitive threshold studies focus on performance on a single course or within a major. Given the significance of finding a cognitive threshold for our understanding of the relationship between cognitive ability and academic performance, as well as the small number of studies on the topic (Hsu and Schombert 2010; Tynan et al. 2020), we hope to further investigate the possibility of cognitive thresholds through replicating Hsu and Schombert’s findings on the mathematics and physics majors, and then by expanding the focus to a widely known very difficult course, organic chemistry.

The results from the two studies indicate that there does not seem to be a clear-cut and absolute cognitive threshold point below which students are doomed to fail highly quantitative courses regardless of the amount of effort they devote into learning. As we discussed earlier, not finding a threshold could be a result of the compensatory effect of non-intellective factors, such as motivation, study habits, and self-selection, such that students with a low SAT math score choose not to take highly quantitative classes or drop out half-way when sensing they are not doing well in the class. Especially given the fact that we only considered students who survived through the whole four-year study or the whole course (i.e., students who still majored in mathematics and physics at their fourth year of college or students who completed organic chemistry), it is very likely that students with a low level of quantitative ability had already left the major/course. However, despite the absence of an absolute cognitive threshold for quantitative courses, we did find that the chance of students with a low level of quantitative ability succeeding in highly quantitative courses is very small, which might qualify for the virtually necessary condition proposed by Dul (2016). Moreover, the present study offers a demonstration of NCA to examine the constraining effect of quantitative reasoning on grades in courses with a quantitative loading, and future studies could employ this method to investigate the more direct necessary-but-not-sufficient conditions in academic settings, such as the relationship between grades in courses that require prerequisites and the performance in prerequisite courses. Additionally, researchers could conduct more exploration on course and student characteristics that can influence NCA results.

Lastly, it is worth noting several shortcomings of NCA. First, as an analytical method that was recently developed, NCA and its R package do not produce an error estimate of its *d* statistic, making it hard to assess the influence of sampling error on study results, especially when a small sample is used. Second, its underlying mechanism suggests that NCA results are closely related to the correlation between the two focal variables. For instance, if variables X and Y are uncorrelated with each other, data points will scatter around the mean value of X and spread over the plot, leaving little empty space in the upper left corner of the plot, and thus a small *d* value from NCA. Therefore, it is conceivable that the degree of necessity of X on Y found by NCA is closely related to the correlation between the two variables. We do note that any arguments that NCA does not delve beyond what can be achieved by simply knowing the correlation between two variables would only apply to settings in which assumptions underlying the correlation coefficient are met, specifically, bivariate normality and homogeneity of variance. Mechanisms underlying the presence of necessary conditions for attaining certain levels of the outcome variance in interest are also likely to result in departures from homogeneity of variance (e.g., at high levels of the X variables of interest, all levels of Y are possible, but at lower levels the attainable levels of Y are constrained). Thus, NCA can provide information above that obtained via a correlation coefficient. We also note that NCA does offer an effect size to help quantify the extent to which the independent variable imposes a constraining effect on the dependent variable, and it also provides a visualization that illustrates the effect clearly. Therefore, we view NCA as a useful tool for some purposes.

## 9. Implications

The findings from the two studies imply that, for courses that require a high level of quantitative ability, such as those taken by math- and physics-majored students and in organic chemistry, starting with a relatively low level of quantitative ability greatly disadvantages students and makes it very unlikely for them to obtain a satisfactory grade at the end of their study. Therefore, depending on their levels of quantitative ability, students should be judicious of their major choice and take into consideration their past records on quantitative performance, especially if they aspire to enter into highly quantitative majors, such as mathematics and physics. Additionally, college class advisors should be very careful in recommending students with low SAT math scores to take highly quantitative classes such as organic chemistry, as the chance of them succeeding in the class is rather slim. Although a small percentage of students with low quantitative ability had completed their courses with a high grade, it is not clear whether this is because these students worked especially hard for their grades, their professors were more lenient graders, or because they initially did not perform to the best of their ability on the SAT math exam. Previous studies have found that even the selection of organic chemistry textbooks could have a significant effect upon students’ course success (Houseknecht 2010). Therefore, results related to these groups of students should be interpreted with caution.

## 10. Conclusions

Through two studies, we conducted a thorough investigation of the existence of cognitive thresholds in highly quantitative courses using a multi-institutional dataset, and we did not find such a point on the quantitative ability continuum below which the chance of students succeeding on these courses goes to zero. Therefore, students and college course advisors should take into consideration the student’s previous records in relevant domains when selecting courses for enrollment.

## Figures and Tables

**Table 1:** Percentages of students in each combination of SAT math scores and course grades in organic chemistry class.

SAT Math Scores	Total *N*	Course Grade			
		B (3)	B+ (3.1–3.5)	A− (3.6–3.9)	A (4)
<400	277	10%	1%	1%	3%
400–500	2906	15%	4%	2%	6%
500–600	12,843	19%	5%	3%	10%
600–700	21,050	23%	8%	6%	17%
700–800	11,114	22%	8%	9%	28%

*Note.* SAT math scores range from 200 to 800. Course grade ranges from F (0) to A (4).

## Data Availability

Datasets used in this study is not publicly available as they were supported by a grant from the College Board.

## Note

1 Free disposal hull is the label used by Dul (2016) to indicate that there are not any assumptions regarding the ceiling line.
